# Association of basal serum testosterone levels with ovarian response and in vitro fertilization outcome

**DOI:** 10.1186/1477-7827-9-9

**Published:** 2011-01-20

**Authors:** Yingying Qin, Zhiyi Zhao, Mei Sun, Ling Geng, Li Che, Zi-Jiang Chen

**Affiliations:** 1 Center for Reproductive Medicine, Shandong Provincial Hospital, Shandong University, Jinan, 250021, PR China; 2 Key Laboratory of Reproductive Medicine, Shandong Province, PR China; 3 Case Western Reserve University, Cleveland, OH 44146, USA

## Abstract

**Background:**

To evaluate basal testosterone (T) levels during follicular phase of the menstrual cycle as a predictor for ovarian response and in vitro fertilization (IVF) outcome.

**Method:**

We analyzed data retrospectively from hospital-based IVF center including one thousand two hundred and sixty Chinese Han women under their first IVF cycle reached the ovum pick-up stage, without polycystic ovary syndrome (PCOS) or endometriosis undergoing long IVF protocol. Patients were divided into 2 groups. Group 1: patients with diminished ovarian reserve (basal FSH >10 IU/L) (n = 187); Group 2: patients with normal ovarian reserve (basal FSH < = 10 IU/L) (n = 1073). We studied the association of basal T levels with ovarian response and IVF outcome in the two groups. Long luteal down-regulation protocol was used in all patients, that is, the gonadotropin releasing hormone agonist was administered in the midluteal phase of the previous cycle and use of recombinant FSH was started when satisfactory pituitary desensitization was achieved.

**Results:**

Basal T levels were markly different between pregnant and non-pregnant women in Group 1; whereas not in Group 2. A testosterone level of 47.85 ng/dl was shown to predict pregnancy outcome with a sensitivity of 52.8% and specificity of 65.3%; and the basal T was correlated with the numbers of large follicles (> 14 mm) on HCG day in Group 1. Significantly negative correlations were observed between basal T, days of stimulation and total dose of gonadotropins after adjusting for confounding factors in both groups.

**Conclusion:**

In women with diminished ovarian reserve, basal T level was a predictor for the number of large follicles on HCG day and pregnancy outcome; but could not in those with normal serum FSH. Basal T levels were associated with both days of stimulation and total dose of gonadotropins, indicating that lower level of T might relate with potential ovarian poor response.

## Background

Ovarian response, the recruitment and development of multiple follicles followed by gonadotropins, is a key factor for in vitro fertilization (IVF) treatment cycle. The prediction of ovarian response before undertaking the expensive IVF treatment is important. However, the predictive value of various widely used markers, such as age, antral follicle count (AFC), levels of serum inhibin B, serum anti-Müllerian hormone (AMH), basal serum follicle stimulating hormone (FSH) and estradiol (E_2_), basal FSH/LH (luteinizing hormone) ratio, still appeared inconsistent and not accurate enough. A large amount of patients may respond poorly to gonadotropins in spite of normal screening parameters [1]. Therefore, it continues to be a challenge for reproductive physicians to identify poor ovarian reserve and the probability of pregnancy beforehand.

Androgens, primarily testosterone (T) and androstenedione, are noteworthy to enhance follicular recruitment [2], promote follicular growth and development [3,4], and increase insulin like growth factor 1 (IGF-1) expression in the primate ovary which plays an essential role in regulating follicular development [3,5]. Recent clinical reports with encouraging results demonstrated that co-treatment with androgen, such as dehydroepiandrosterone (DHEA) and Androderm (transdermal testosterone), could increase both quantity and quality of oocytes and embryos, and improve pregnancy outcomes in women with diminished ovarian function or even premature ovarian failure [6-9]. Improved controlled ovarian hyperstimulation (COH) and pregnancy outcomes by supplementation with aromatase inhibitors may be the consequence of increased intraovarian androgen, followed by the induction of FSH receptors on granulosa cell [10,11]. Nevertheless, controversy exists as to whether these protocols improve cycle outcome [12,13].

Prompted by these findings, we hypothesized whether serum testosterone levels could predict ovarian response and IVF outcome. To the best of our knowledge, only a few studies have concerned this question with conflicting results. This might be explained by limited sample size and heterogeneous population mixed by patients with polycystic ovary syndrome (PCOS) and/or endometriosis as PCOS and endometriosis are associated with higher or lower serum T levels [14,15]. No published study focus on the role of basal T levels during stimulation cycle on the ovarian response and reproductive outcomes in women with either diminished or normal ovarian reserve. Thus, conclusive and definite data about the associations of basal serum T levels with ovarian response and IVF outcome are still lacking. The purpose of the present study is to evaluate the predictive value of basal T levels in women with both diminished and normal ovarian reserve on ovarian response and IVF outcome in a large (n = 1260) and homogeneous population undergoing IVF-ET.

## Methods

### Study population

One thousand two hundred and sixty Chinese Han patients under their first IVF-ET cycle reached the ovum pick-up stage undergoing IVF in the Center for Reproductive Medicine, Shandong Provincial Hospital, Shandong University from October, 2008 to January, 2010 were recruited. And we selected women who used only long protocol to avoid possible bias from different protocols. Exclusion criteria were women with either PCOS or endometriosis. Patients were divided into 2 groups. Group 1 included patients with diminished ovarian reserve (n = 187) and Group 2 with normal ovarian reserve (n = 1073). Diminished ovarian reserve was defined as basal FSH value >10 IU/L [13]. We studied the association of basal T levels with ovarian response and IVF outcome in the two groups. Informed consent was obtained from all subjects. This study received institutional review board approval from the ethical committee of Shandong University.

### Study protocol

Long luteal down-regulation protocol was used in all patients. Briefly, the gonadotropinreleasing hormone (GnRH) agonist (0.1 mg/ampoule, Tryptorelin, Ferring, Germany) was administered in the midluteal phase of the previous cycle until the day of HCG administration. After onset of menstrual bleeding, when satisfactory pituitary desensitization was achieved (serum E2 level was lower than 50 pg/ml), gonadotropin stimulation was started with a daily use of recombinant FSH (75 U/ampoule, Gonal F, Serono. Ltd, Switzerland). The dose of gonadotropins was adjusted according to follicular growth monitored by transvaginal ultrasonography and serum E2 concentrations. HCG (10,000 IU) (Lizhu. Ltd, Guangdong, China) was given when at least two follicles reached 18 mm in diameter and 36 hours later oocytes were retrieved by transvaginal ultrasound-guided follicular aspiration. Up to three embryos per patient were transferred (2 embryos were transferred for women aged <35 years old and in their first treatment cycle, 3 embryos were transferred for women aged ≥ 35 years old or ≥ 2 treatment cycles) 2 or 3 days after oocytes retrieval and the luteal phase was supported with progesterone (20 mg/ampoule, Xianju Ltd, Zhejiang, China). A positive pregnancy was defined by > 5 IU/L of plasma β-hCG on 14 days after embryo transfer. The fertilization rate was defined as the number of oocytes with two pronuclei after insemination divided by the number of oocytes inseminated. The spontaneous miscarriage was defined as both biochemical and clinical miscarriages. The good quality embryos were defined as embryos developed from normal fertilized eggs with no fragmentation or no more than 1/3, no presence of multinucleation, 3~5 blastomeres at 48 hours after egg retrieval and at least 7 blastomeres by 72 h [16].

### Hormone assays

On day 2 to 4 of the menstrual cycle, basal serum levels of T, FSH, E_2 _and LH were obtained from each subject. All the serum levels were determined by the electrochemiluminescent immunoassay in the analyzer cobas e 601 (Roche, Switzerland). The analytical sensitivity of basal T, FSH, LH, E_2 _and β-hCG levels was 0.42 ng/ml, < 0.10 mIU/ml, 0.10 mIU/ml, 5.0 pg/ml and < 0.1 mIU/ml, respectively.

### Statistical analysis

The pretreatment parameters included age, body mass index (BMI), pregnancy history, AFC, starting doses of gonadotropins and basal levels of FSH, E_2_, LH, and T.

To assess the predictive value of basal T levels to the ovarian response, we studied the associations of basal T levels with the number of oocytes retrieved, number of follicles >14 mm on HCG day, basal FSH/LH ratio, days of stimulation and total dose of gonadotropins.

To evaluate IVF outcome, we analyzed the following variables: endometrial thickness on HCG day, fertilization rate, number of embryos cryopreserved, number of good quality embryos, pregnancy and spontaneous miscarriage outcome and live births.

Student's t-test and analysis of covariance (ANCOVA) were performed to examine the differences in means between the pregnant group and the non-pregnant group. Pearson correlation tests and partial correlation analyses were used to assess the relationship between basal T levels and the continuous parameters. Multiple linear regression analyses were used to find the possible independent determinants of test variables among those parameters showing significant correlations and their contributions. The area under the receiver operating characteristic curve (ROC) was performed to examine the predictive value for pregnancy and spontaneous miscarriage outcome. Data were analyzed with SPSS statistical package (SPSS version 13.0, Chicago, IL). Continuous values were presented as mean ± SD and all tests were two-tailed. P < 0.05 was considered statistically significant.

## Results

Table 1 showed the comparison of characteristics and COH parameters between pregnant and non-pregnant (excluded patients who did not have embryo transferred) women in Group 1 and 2. In Group 1, basal T levels were significantly higher in the pregnant women than that in non-pregnant ones (p < 0.05), while as not in Group 2. When adjusting for age yielded similar results. It seemed that pregnant women in Group 1 had more good quality embryos, embryos cryopreservered and more gonadotropins used than non-pregnant patients.

**Table 1:** The comparison of characteristics and COH parameters between pregnant and non-pregnant women

	Group 1			Group 2		
Variables	Non-pregnant cycles (n = 89)	Pregnant cycles (n = 72)	P value	Non-pregnant cycles (n = 403)	Pregnant cycles (n = 518)	P value
Age (years)	32.2 ± 4.0	32.7 ± 3.8	0.352	31.9 ± 4.0	31.1 ± 4.1	0.003
BMI (kg/m^2^)	22.4 ± 2.9	2.2 ± 2.6	0.696	9.4 ± 1.5	9.4 ± 1.4	0.312
Pregnancy history	0.6 ± 0.5	0.6 ± 0.5	0.628	9.0 ± 3.9	9.0 ± 3.8	0.930
Basal FSH levels (IU/L)	11.8 ± 1.9	11.6 ± 1.7	0.424	7.5 ± 1.4	7.5 ± 1.4	0.947
Basal LH levels (IU/L)	5.4 ± 1.9	5.0 ± 2.1	0.260	4.3 ± 2.2	4.3 ± 2.2	0.974
Basal E_2 _levels (pg/ml)	43.3 ± 20.8	41.6 ± 18.3	0.581	43.3 ± 30.5	41.2 ± 31.4	0.300
Basal T levels (ng/dl)	48.3 ± 17.3	40.8 ± 18.1	0.008	47.8 ± 18.9	47.7 ± 19.9	0.907
Antral follicle count	9.8 ± 4.3	8.6 ± 4.6	0.099	11.6 ± 5.5	11.2 ± 5.7	0.217
Starting dose of gonadotropins (IU)	223.3 ± 54.6	228.1 ± 67.3	0.617	203.6 ± 36.7	198.6 ± 40.7	0.053
Number of oocytes retrieved	10.0 ± 4.4	9.0 ± 4.7	0.171	12.4 ± 5.4	12.4 ± 5.3	0.999
Number of follicles >14 mm	7.8 ± 3.5	7.1 ± 3.5	0.176	9.0 ± 3.9	9.0 ± 3.8	0.761
Days of stimulation	9.7 ± 1.6	10.5 ± 1.9	0.002	9.4 ± 1.5	9.4 ± 1.4	0.744
Total dose of gonadotropins (IU)	2477.8 ± 873.9	2936.1 ± 1249.9	0.010	2174.2 ± 826.8	2042.3 ± 728.7	0.012
Endometrial thickness on HCG day (cm)	1.1 ± 0.2	1.1 ± 0.2	0.521	1.0 ± 0.2	1.1 ± 0.2	0.001
Fertilization rate	0.8 ± 0.2	0.7 ± 0.2	0.202	0.7 ± 0.2	0.7 ± 0.2	0.615
Number of embryos cryopreserved	3.3 ± 2.9	2.4 ± 2.4	0.034	3.9 ± 3.4	4.1 ± 3.2	0.256
Number of good quality embryos	5.6 ± 2.9	4.5 ± 2.5	0.015	6.0 ± 3.4	6.3 ± 3.2	0.219

Note: Data other than P values are mean ± SD.

Dichotomy variable pregnancy history was represented by secondary infertility = 1 and primary infertility = 0.

In Group 1, pearson rank correlation analysis showed positive associations between basal T levels with the number of follicles >14 mm on HCG day, but negative associations with age, days of stimulation and total dose of gonadotropins used. When adjusting for age yielded similar results (Table 2). In the pearson rank correlation analysis, the characteristics associated with number of follicles >14 mm on HCG day were age, BMI, AFC, basal T levels, basal FSH levels, basal LH levels and starting dose of gonadotropins, respectively (data not shown). For total dose of gonadotropins, the associated characteristics were age, BMI, AFC, basal T levels, basal FSH levels and starting dose of gonadotropins, respectively (data not shown). And the characteristics correlated with days of stimulation were basal T levels and AFC (data not shown). Accordingly, multiple linear regression analysis were performed to study the major independent factors for number of follicles >14 mm on HCG day, total dose of gonadotropins and days of stimulation which were used as the dependent variable, whereas independent variables included the characteristics showing significant correlations mentioned above respectively (Table 3, 4, 5). Basal T level was an independent factor for number of follicles >14 mm on HCG day, total dose of gonadotropins and days of stimulation. For number of follicles >14 mm on HCG day, the influencing independent factor with decreasing importance were basal FSH levels, AFC, basal LH levels, basal T levels and age. For total dose of gonadotropins, the largest influencing independent factor was starting dose of gonadotropins, and with decreasing importance, basal FSH levels and basal T levels in order. For days of stimulation, the only influencing independent factor was basal T levels.

**Table 2:** Spearman correlation analysis of associations between basal T levels and the characteristics and COH parameters

	Group 1				Group 2			
Variables	r value	P value	**r value**^**a**^	**P value**^**a**^	r value	P value	**r value**^**b**^	**P value**^**b**^
Age (years)	-0.163	0.026	-	-	-0.151	<0.001	-	-
BMI (kg/m^2^)	-0.054	0.308	-0.009	0.907	-0.063	0.038	-	-
Pregnancy history	-0.031	0.669	0.013	0.859	-0.033	0.276	0.011	0.714
Basal FSH level (IU/L)	-0.051	0.068	-0.032	0.664	0.028	0.361	0.033	0.281
Basal LH level (IU/L)	0.026	0.722	0.007	0.927	0.080	0.009	-	-
Basal E_2 _level (pg/ml)	0.141	0.054	0.134	0.069	-0.044	0.150	-0.036	0.239
Antral follicle count	0.026	0.724	0.002	0.979	0.044	0.151	0.015	0.631
Starting dose of gonadotropins (IU)	-0.088	0.434	-0.009	0.902	-0.124	<0.001	-	-
Basal FSH/LH ratio	-0.014	0.849	0.013	0.856	-0.056	0.065	0.006	0.839
Numbers of oocytes retrieved	0.115	0.117	0.063	0.394	0.064	0.037	0.009	0.766
Number of follicles >14 mm	0.191	0.009	0.147	0.047	0.013	0.679	-0.036	0.246
Days of stimulation	-0.174	0.017	-0.177	0.016	-0.076	0.013	-0.066	0.031
Total dose of gonadotropins (IU)	-0.203	0.005	-0.155	0.035	-0.139	<0.001	-0.081	0.008
Endometrial thickness on HCG day (cm)	0.120	0.102	0.093	0.211	-0.027	0.380	-0.041	0.177
Fertilization rate	-0.010	0.893	-0.007	0.930	-0.007	0.828	0.003	0.922
Number of embryos cryopreserved	0.069	0.347	0.020	0.791	0.058	0.058	0.009	0.766
Number of good quality embryos	0.060	0.412	0.022	0.771	0.040	0.190	0.002	0.956
Live births	-0.009	0.906	-0.022	0.765	-0.024	0.427	-0.023	0.452

Note: ^a ^represent the r value and P value after adjusting for age that associated with basal testosterone levels.

^b ^represent the r value and P value after adjusting for age, basal LH level, BMI and starting dose of gonadotropins that associated with basal testosterone levels.

Dichotomy variable pregnancy history was represented by secondary infertility = 1 and primary infertility = 0.

**Table 3:** Multiple linear regression analysis of possible determinants for number of follicles >14 mm in Group 1

	Coefficients				
	Unstandardized coefficients		Standardized coefficients		
Independent variables	β	Std. Error	β	t	P value
(constant)	15.654	3.387		4.622	<0.001
Age (years)	-0.151	0.074	-0.157	-2.027	0.044
BMI (kg/m^2^)	-0.092	0.100	-0.066	-0.915	0.361
Basal T levels (ng/dl)	0.028	0.014	0.136	2.032	0.044
Basal FSH level (IU/L)	-0.414	0.126	-0.227	-3.288	0.001
Basal LH level (IU/L)	0.330	0.134	0.175	2.459	0.015
Antral follicle count	0.150	0.054	0.186	2.777	0.006
Starting dose of gonadotropins (IU)	-0.003	0.005	-0.047	-0.624	0.534

Note: The dependent variable is number of follicles >14 mm. The model (r = 0.475, r^2 ^= 0.225, adjusted r^2 ^= 0.195, P < 0.001).

The values of the standardized coefficients reflect the independent contributions of each predictor to dependent variables.

**Table 4:** Multiple linear regression analysis of possible determinants for total dose of gonadotropins in Group 1

	Coefficients				
	Unstandardized coefficients		Standardized coefficients		
Independent variables	β	Std. Error	β	t	P value
(constant)	-464.984	819.634		-0.567	0.571
Age (years)	-8.988	18.659	-0.032	-0.482	0.631
BMI (kg/m^2^)	40.781	25.082	0.098	1.626	0.106
Basal T levels (ng/dl)	-8.833	3.516	-0.145	2.512	0.013
Basal FSH level (IU/L)	89.904	31.525	0.166	2.852	0.005
Antral follicle count	-26.244	13.797	-0.109	-1.902	0.059
Starting dose of gonadotropins (IU)	9.633	1.168	0.531	8.244	<0.001

Note: The dependent variable is total dose of gonadotropins. The model (r = 0.647, r^2 ^= 0.419, adjusted r^2 ^= 0.399, P < 0.001).

The values of the standardized coefficients reflect the independent contributions of each predictor to dependent variables.

**Table 5:** Multiple linear regression analysis of possible determinants for days of stimulation.

	Coefficients				
	Unstandardized coefficients		Standardized coefficients		
Independent variables	β	Std. Error	β	t	P value
(constant)	11.388	0.427		26.661	<0.001
Basal T levels (ng/dl)	-0.017	0.007	-0.170	-2.367	0.019
Antral follicle count	-0.054	0.028	-0.141	-1.964	0.051

Note: The dependent variable is days of stimulation. The model (r = 0.224, r^2 ^= 0.050, adjusted r^2 ^= 0.040, P = 0.009).

The values of the standardized coefficients reflect the independent contributions of each predictor to dependent variables.

Table 6 shows the ROC curve analysis of the diagnostic accuracy of discriminination between pregnancy per transfer versus non-pregnancy per transfer, spontaneous miscarriage and non-spontaneous miscarriage. In the ROC model, basal T levels are predictive of pregnancy outcome in Group 1. A basal T level of 47.85 ng/dl was shown to predict pregnancy outcome with a sensitivity of 52.8% and specificity of 65.3%.

**Table 6:** Receiver operating characteristic curve analysis of study variables for the prediction of IVF outcome.

	Variables	**AUC**_**ROC**_	Optimum cutoff	Sensitivity %	Specificity %	95% CI	P value
Group 1	Pregnancy per transfer	0.609	47.85	52.8	65.3	0.521-0.696	0.018
	Spontaneous miscarriage	0.396	18.24	100	4.1	0.245-0.548	0.196
Group 2	Pregnancy per transfer	0.508	47.26	52.3	52.9	0.470-0.546	0.673
	Spontaneous miscarriage	0.505	54.76	40.0	67.4	0.441-0.569	0.871

Note: AUC_ROC _= area under the receiver operating characteristic curve; CI = confidence interval.

## Discussion

Androgen actions via the AR play an important role in female fertility [17,18]. It is believed that AR signaling in granulosa cells (GCs) is necessary for normal follicular growth and progression beyond preantral stage [19]. Inhibition of ARs slows mouse follicle growth [20], decreases the diameter of mouse follicles [21,22] and induces GC degeneration and follicular apoptosis. Low numbers of antral follicles, ovulated oocytes, and a high rate of follicular atresia were found in the global and GC-specific ARKO mice [23,24]. In this study, basal T level in women with diminished ovarian reserve positively correlates with the number of large follicles (>14 mm) significantly. This is in agreement with previous studies mentioned above.

With IVF being an invasive, stressful, time-consuming and expensive treatment, it is important to help couples select the best individualized strategy for COH. Predicting the ovarian response is useful in ameliorating subsequent disappointment and distress of potential poor responders as well as in optimizing the gonadotropins stimulation protocol to obtain a good response. Although no significant association between basal T levels and oocytes retrieved was found, basal T level is an independent factor for both days of stimulation and total dose of gonadotropins in both groups. In other words, in order to reach similar ovarian response, patients with lower basal T levels needed more days and more doses of gonadotropins otherwise they might show poor ovarian response.

Basal FSH/LH ratio is suggested to predict ovarian response and IVF outcome, although the definite threshold value is still controversial [25,26]. We assessed the correlation of basal FSH/LH ratio and basal T level for the first time in the present study and found no significant association in the two groups. Further prospective studies are needed to elucidate the subject.

The application of basal serum T levels for predicting pregnancy outcome is debated. John L et al. suggested basal testosterone levels ≤ 20 ng/dL was associated with poor IVF success rates [27]. But later, the same group and Barbieri RL denied the predictive value in other studies [15,28]. In this study, we showed for the first time that basal T level could predict pregnancy outcome in women with diminished ovarian reserve. We compared the relative performance of basal T level with basal FSH, basal E2 and AFC using ROC curve further as a predictor of pregnancy outcome in Group 1. Only basal T levels was a significant indicator for pregnancy outcome. There is no report focusing on the relationship between basal T level and spontaneous miscarriage outcome at present. Our results showed that, on pregnancy outcome, basal T levels had minimal effects, and for the first time, no association with spontaneous miscarriage. As is known to all, a successful IVF treatment is dependent on ovarian response, as well as oocyte quality, male factors and endometrial factors. Although basal T level is associated with potential ovarian response in women with normal ovarian reserve according to the present study, it could not predict the pregnancy outcome as expected.

A weakness of this study is only basal serum testosterone level, which is ovarian androgen, was evaluated. DHEA and other adrenal androgens are also very important as shown in previous studies [6,29]. Further prospective investigations on adrenal androgens are warranted in prospective studies.

One strength of our study is the largest sample size (total 1260 patients) so far. Secondly, different from previous studies [15,27,28], the population we investigated was excluded PCOS and endometriosis. Different kinds of protocols used other than long protocol was ruled out. And we adjusted for confounding variables including age, BMI, treatment cycles, infertility history and starting dose of gonadotropins given their possible associations with basal serum T levels. It is reported that age, BMI and causes of infertility may involve in ovarian response and/or pregnancy outcome [30-32]. Therefore, patients in our study were matched for these variables, allowing the analysis of basal T levels as an independent marker for test variables. Besides, although this is a retrospective study, ascertainment and recall bias were minimized as all the data were collected and recorded in the computerized database.

## Conclusions

Basal T level is a good predictor for pregnancy outcome and number of large follicles on HCG day in women with diminished ovarian reserve. Basal T level is equally helpful in tailoring the dosage of gonadotropins to individual and identifying potential poor ovarian responders, subsequently, making individualized COH strategy before entering IVF cycles. It also gives evidence to androgen supplementation in infertile women. Those patients with lower basal T levels would benefit from T supplementation during COH, such as improving response, decreasing the amount of gonadotropins used and the cost accordingly.

## Abbreviations

IVF: in vitro fertilization; PCOS: polycystic ovary syndrome; AFC: antral follicle count; AMH: anti-Müllerian hormone; FSH: follicle stimulating hormone; E_2_: estradiol; IGF-1: insulin like growth factor 1; COH: controlled ovarian hyperstimulation; GnRH: gonadotropinreleasing hormone; T: testosterone; ANCOVA: analysis of covariance; DHEA: Dehydroepiandrosterone; GCs: granulosa cells.

## Competing interests

The authors declare that they have no competing interests.

## Authors' contributions

ZC designed the study and supervised patients' diagnosis, samples recruitment and experiments performance. ZZ conducted data analysis and drafted the manuscript. MS and LG recruited samples. YQ revised the manuscript, performed statistical analysis and endocrine biochemical examination. LC revised the manuscript. All authors read and approved the final manuscript.
